# Bacteriophage- based tests for the detection of *Mycobacterium tuberculosis *in clinical specimens: a systematic review and meta- analysis

**DOI:** 10.1186/1471-2334-5-59

**Published:** 2005-07-16

**Authors:** Shriprakash Kalantri, Madhukar Pai, Lisa Pascopella, Lee Riley, Arthur Reingold

**Affiliations:** 1 University of California, Berkeley, School of Public Health, 140, Warren Hall, Berkeley, CA 94720, USA; 2 Mahatma Gandhi Institute of Medical Sciences, Sevagram, Maharashtra, India; 3 Division of Pulmonary & Critical Care Medicine, San Francisco General Hospital, San Francisco, CA 94110, USA; 4 Surveillance and Epidemiology Section, Tuberculosis Control Branch, California Department of Health Services, Berkeley, CA, USA

## Abstract

**Background:**

Sputum microscopy, the most important conventional test for tuberculosis, is specific in settings with high burden of tuberculosis and low prevalence of non tuberculous mycobacteria. However, the test lacks sensitivity. Although bacteriophage-based tests for tuberculosis have shown promising results, their overall accuracy has not been systematically evaluated.

**Methods:**

We did a systematic review and meta-analysis of published studies to evaluate the accuracy of phage-based tests for the direct detection of *M. tuberculosis *in clinical specimens. To identify studies, we searched Medline, EMBASE, Web of science and BIOSIS, and contacted authors, experts and test manufacturers. Thirteen studies, all based on phage amplification method, met our inclusion criteria. Overall accuracy was evaluated using forest plots, summary receiver operating (SROC) curves, and subgroup analyses.

**Results:**

The data suggest that phage-based assays have high specificity (range 0.83 to 1.00), but modest and variable sensitivity (range 0.21 to 0.88). The sensitivity ranged between 0.29 and 0.87 among smear-positive, and 0.13 to 0.78 among smear-negative specimens. The specificity ranged between 0.60 and 0.88 among smear-positive and 0.89 to 0.99 among smear-negative specimens. SROC analyses suggest that overall accuracy of phage-based assays is slightly higher than smear microscopy in direct head-to-head comparisons.

**Conclusion:**

Phage-based assays have high specificity but lower and variable sensitivity. Their performance characteristics are similar to sputum microscopy. Phage assays cannot replace conventional diagnostic tests such as microscopy and culture at this time. Further research is required to identify methods that can enhance the sensitivity of phage-based assays without compromising the high specificity.

## Background

Tuberculosis (TB) is a leading cause of morbidity and mortality worldwide. According to the World Health Organization, about one-third of the world's population is infected with *Mycobacterium tuberculosis*, and about 8 million new cases of TB occur each year. Despite this large burden and intensive control efforts, only about 46% of the new infectious TB cases are detected each year [1].

Conventional TB diagnostics include sputum microscopy and culture of *M. tuberculosis*. Although microscopy is simple, specific and rapid, it suffers from low sensitivity (30–70%) [2]. Microscopy is particularly insensitive in HIV-infected populations. The laboratory turn around time for *M. tuberculosis *growth on solid culture media is around eight weeks. Cultures on liquid media are more rapid. Although the cost of liquid cultures has recently been reduced, most hospitals in developing countries may not find the test affordable. Low-income countries needs diagnostic tools that are sensitive, specific, cost effective, easy to perform, and easy to implement within the current infrastructure [3].

Among the various alternative diagnostic tests being evaluated, tests based on mycobacteriophages have shown promise [4]. Phage-based tests are relatively easy to perform, but requires the type of laboratory infrastructure that is needed for routine mycobacterial cultures. The turnaround time of phage-based tests is 2 days compared to about 2 hours (microscopy) or up to 2 months (culture). Two large-scale studies have shown that the test detected 65–83% of the confirmed TB cases within 48 hours; the specificity of the tests in each of the studies was >95% [5,6]. The assay could offer new tools for the rapid diagnosis of TB in both the developed and developing world [3-18]. However, it is important to systematically review the literature and summarize the current evidence on these new assays.

**Figure 1:**
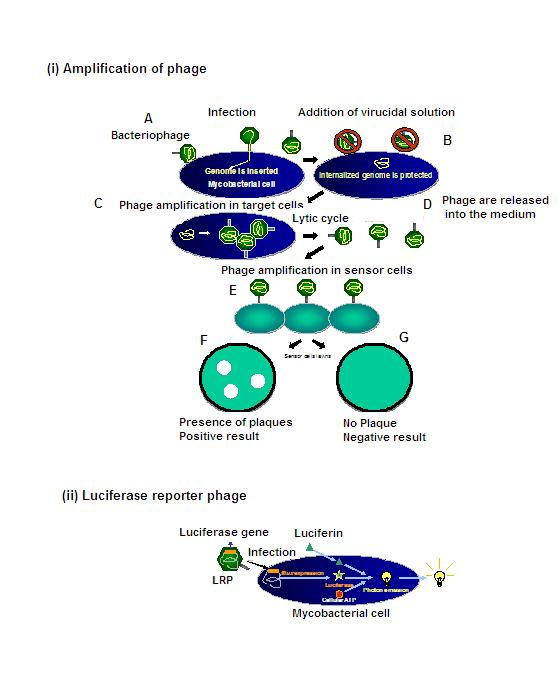
Bacteriophage-based assays for diagnosis of tuberculosis.

Two main phage-based approaches are used to detect *M. tuberculosis *(Figure 1) [see Additional file]: (i) amplification of phages after their infection of *M. tuberculosis*, followed by detection of progeny phages using helper cells (plaque formation); and (ii) detection of light produced by luciferase reporter phages (LRP) by live *M. tuberculosis *[3]. Phage-based tests are available as commercial kits (e.g. FASTPlaque-TB^® ^and PhageTek MB^®^, a name variant of the FASTPlaque-TB, Biotec Laboratories Ltd, UK) [14] and as in-house (laboratory-developed) assays [15]. In-house tests use either amplification technology (e.g. phage amplified biologically [PhaB]) or LRPs. Some of the phage-based tests are designed to rapidly detect rifampin resistance (e.g. FASTPlaque-MDRi) in culture isolates. Existing studies show that the accuracy of phage based tests might vary [18]. Because traditional reviews are often subjective, less comprehensive, and rely on qualitative methods [19], we conducted a systematic review to evaluate the overall accuracy of phage-based tests for the direct detection of *M. tuberculosis *in clinical specimens. We addressed the following questions in our review:

1. What is the overall accuracy of phage-based tests compared to the gold standard, culture?

2. What is the overall accuracy of phage-based tests compared to smear microscopy?

3. How does the accuracy of phage-based tests vary by sputum smear status?

## Methods

### Search strategy and selection criteria

We searched the following electronic databases: PubMed (1985–2004), Web of Science (1985–2004), EMBASE (1988–2004), and BIOSIS (1993–2004). All searches were up to date as of November 2004. The search terms included "tuberculosis," "Mycobacterium tuberculosis," "mycobacteria," "bacteriophages," "mycobacteriophage," "phage," "FASTPlaque," "phage amplification," "phage-based," "bacteriophage-based," "sensitivity and specificity", "accuracy" and "predictive value". We also searched the reference lists from the primary studies and review articles, sought help from experts in the field, and obtained lists of studies from companies that manufacture commercial rests. Although we did not impose language restriction while searching, we included only English language articles for our review.

We followed a written protocol and explicit study selection criteria. Studies were included in the review if they met the following criteria: comparison of phage test against a reference standard, data necessary for the computation of both sensitivity and specificity, and culture (either liquid or solid media) as the reference standard. Two reviewers (SK and MP) independently screened the titles and abstracts to identify eligible studies. Disagreements between the reviewers were resolved by consensus. A list of excluded studies, along with the reasons for exclusion is available from the authors on request.

### Data abstraction and quality assessment

The final set of included articles was assessed by one reviewer (SK), who extracted data from all the studies using a piloted data extraction form. A second reviewer (MP) independently extracted data from a subset of the included studies to evaluate reproducibility of data extraction. The inter-rater agreement between the two reviewers was 100%. Data retrieved from the reports included methodological quality, participant characteristics, laboratory methods, and outcome data (sensitivity and specificity). We assessed the quality of the studies by using criteria selected from the QUADAS checklist [20] for assessment of quality of diagnostic studies: study design (cross-sectional versus case-control), blinding (single/double blind versus unblinded interpretation of index test and reference standard results), and potential for verification bias (complete versus partial/differential verification of index test results by reference standards).

To compute sensitivity and specificity of phage-based assays, we chose sputum culture on solid and/or liquid media (e.g. BACTEC 460) as the reference standard in our review. To compare the accuracy of phage based assays with sputum microscopy, we evaluated studies that reported head-to-head comparisons of sputum microscopy with phage based assays (against a common reference standard) and computed the diagnostic yield of phage based assays over and above sputum microscopy. To evaluate the effect of sputum-smear status on accuracy, we conducted subgroup analyses for smear-positive and smear-negative specimens.

### Statistical analysis

We used standard methods recommended for meta-analyses of diagnostic test evaluations [21]. Data were analyzed with Meta-Disc (version 1.1.1) software [22]. Our analyses focused on the following measures of diagnostic accuracy: sensitivity (true positive rate [TPR]), and specificity (1-false positive rate [FPR]).

Each study in the meta-analysis contributed a pair of numbers: TPR and FPR. Since these measures are correlated and vary with the thresholds (cut points), it is customary to analyze TPR and FPR proportions as pairs, and to also explore the effect of threshold on study results [23]. We summarized the joint distribution of sensitivity and specificity using the Summary Receiver Operating Characteristic (SROC) curve. Unlike a traditional Receiver Operating Characteristic plot that explores the effect of varying thresholds on sensitivity and specificity in a single study, each data point in the SROC space represents a separate study. The SROC curve is obtained by fitting a regression curve to pairs of TPR and FPR. The SROC curve and the area under it present an overall summary of test performance, and display the trade off between sensitivity and specificity. A symmetric, shoulder-like SROC curve suggests that variability in thresholds employed could, in part, explain variability in study results [19]. The area under the SROC curve is a global measure of overall test accuracy. An area under the curve of 100% indicates perfect discriminatory ability.

Heterogeneity in meta-analysis refers to a high degree of variability in study results [24]. Such heterogeneity could be due to variability in thresholds, disease spectrum, test methods, and study quality across studies [24]. In the presence of significant heterogeneity, pooled, summary estimates from meta-analyses are not meaningful. We investigated heterogeneity using subgroup analyses. Meta-regression was not attempted because of the small number of studies identified.

## Results

### Study selection

**Figure 2:**
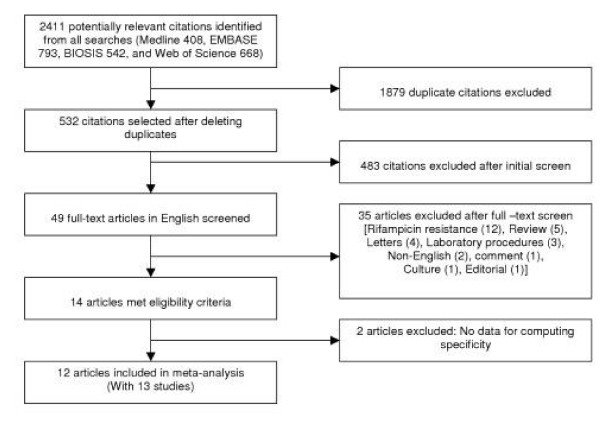
Study flow.

Figure 2 describes the study selection process. Thirteen studies from 12 articles [5-13,15-17], of the 532 articles identified, met our eligible criteria, and were included in our final analyses.

### Description of included studies

Studies included in the final analyses are summarized in Table 1. The total number of samples analysed in all the studies were 5820 (mean 448); 1330 (23%) of the samples were culture positive. Most studies enrolled subjects with suspected pulmonary tuberculosis, as suggested by history, physical examination or chest radiograph. Three studies [8,16,17] included non-respiratory specimens (urine, CSF, pleural fluid and lymph node aspirate], in addition to sputa. Except one [9], no study provided data on age range, sex ratio and prevalence of HIV. In half the studies, patients had received some treatment before they were evaluated. The study design was cross-sectional in 11 of 13 (85%) [5-13,15], and case-control in 2 studies (15%) [11,17]. Only 2 [13,15] of 13(15%) studies reported blinded comparison of the phage based assays with the reference standard. No study had potential for incorporation bias.

**Table 1:** Study characteristics and methodological quality of included studies

**Study Characteristics**	**Frequency**
**Study Design**	
Cross-sectional	11
Case-control	2
**Verification of phage tests with reference standard**	
Complete	13
Partial	0
**Blind assessment of phage tests and reference standard results**	
Yes	3
Unclear	10
**Test before treatment**	
Yes	6
No	5
Unclear	2
**Year of Publication**	
Before 2002	4
After 2002	9
**Study size**	
< 20% positive specimens	2
> 20% positive specimens	11
**Reference standard**	
BACTEC 460	3
LJ medium	6
Bactec and LJ*	3
LJ/AMTD**	1
**Type of assay**	
Commercial	11
In-house	2

*LJ: Lowenstein Jensen medium

**AMTD: Amplified *Mycobacterium tuberculosis *Direct Test

### Assay characteristics

All studies (except one that classified any visible plaques as positive [15] used the same cut point for the phage amplification assay: 20 or more plaques were reported as a positive test. No study used luciferase reporter phages to detect *M. tuberculosis *in clinical specimens. Sputum samples were processed by a standard N-acetyl-L-cysteine-sodium hydroxide (NALC-NaOH) method in all studies evaluating commercial phage-based assays. Only three studies [6,7,13] reported the proportion of the phage tests or the reference tests that were contaminated. Non-tuberculous mycobacteria (NTM) were grown in 4 studies [6,9,10,17], but most studies provided no data on what proportion of the NTM isolates were positive by the phage test. Six studies [6,9,11-13,15] had used LJ cultures as the reference standard; three studies [5,7,10] used BACTEC 460; three studies [8,17] used LJ and BACTEC methods and one study [13] used LJ and AMTD tests. The index test was FASTPlaque in 10 studies [5-8,10-13,16,17], PhageTek in 1 study [9] and in-house amplification assays in 2 studies [13,15]. None of the studies used LRP assays. Most studies reported sensitivity and specificity data with specimens as the unit of analysis (not individuals).

### Overall accuracy of phage assays

**Figure 3:**
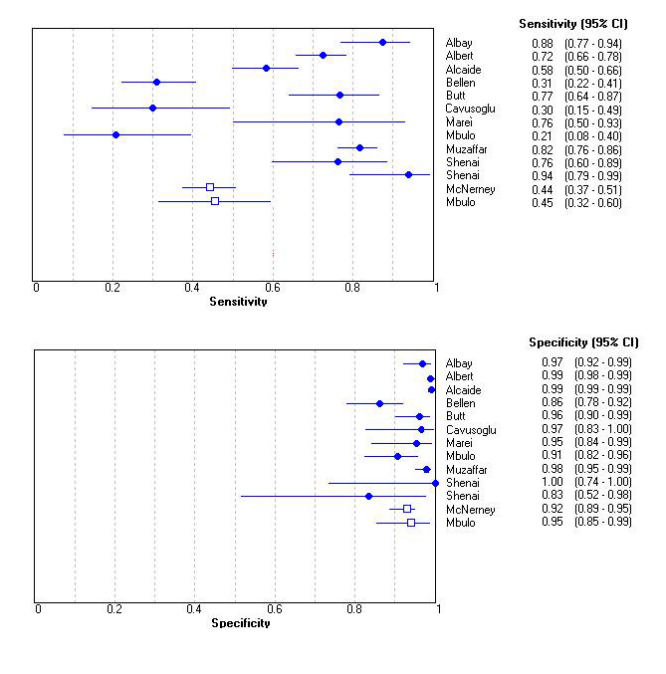
Forest plots of estimates of sensitivity and specificity for commercial and in-house assays. circle: commercial assays; rectangles: in-house assays. Error bars represent 95% CIs.

We identified 13 studies that assessed the sensitivity of phage-based assays (Table 2, Figure 3). Sensitivity is the proportion of patients with culture-proven tuberculosis who are positive by phage-based assays. Overall, as seen in Figure 3, the sensitivity of the phage based assays varied widely, from 0.21 to 0.94 (test of heterogeneity: p < 0.001). Because of the significant heterogeneity, sensitivity estimates were not pooled. Three studies [9,11,13] reported very low sensitivity.

**Table 2:** Description of studies in the meta-analysis and measures of test accuracy

Source	Country	Study Design	Blinding	Complete verification	Specimen	Treatment status	Test	Patients With TB (No./Overall)	Reference Standard	Sensitivity (95%CI)	Specificity (95%CI)
**Commercial assays**											
Albay (2003)	Turkey	CS	Unclear	Yes	Sputum	Untreated	FAST*Plaque*	64/192	BACTEC	0.88 (0.77, .94)	0.97 (0.92, 0.99)
Albert (2002)	South Africa	CS	Unclear	Yes	Sputum	Untreated	FAST*Plaque*	207/1618	BACTEC	0.72 (0.66,0.78	0.99 (0.98,0.99)
Alcaide (2003)	Spain	CS	Unclear	Yes	Sputum+ other	Some treated	FAST*Plaque*	144/2048	B+LJ	0.58 (0.50,0.66)	0.99 (0.99,0.99)
Bellen (2003)	Philippines	CS	Unclear	Yes	Sputum	Some treated	Phage Tek	103/206	LJ	0.31 (0.22,0.41)	0.86 (0.78,0.92)
Butt (2004)	Pakistan	CS	Unclear	Yes	Sputum	Untreated	FAST*Plaque*	60/160	BACTEC	0.77 (0.64,0.87)	0.96 (0.90,0.99)
Cavusoglu (2002)	Turkey	CC	Unclear	Yes	Sputum	Some treated	FAST*Plaque*	33/63	LJ	0.30 (0.15,0.49)	0.97 (0.83,1.00)
Marei (2003)	Egypt	CS	Unclear	Yes	Sputum	Untreated	FAST*Plaque*	60/160	BACTEC	0.77 (0.64,0.87)	0.96 (0.90,0.99)
Mbulo (2004)	Zambia	CS	Yes	Yes	Sputum	Untreated	FAST*Plaque*	29/115	LJ	0.21 (0.08,0.40)	0.91 (0.82,0.96)
Muzaffar (2002)	Pakistan	CS	Unclear	Yes	Sputum	NR	FASTPlaque	103/1209	LJ	0.82 (0.76,0.86)	0.98 (0.95,0.99)
Shenai (2002)	India	CS	Unclear	Yes	Sputum+ other	Some treated	FASTPlaque	62/90	BACTEC+LJ	0.76 (0.60,0.89)	1.00 (0.74,1.00)
Shenai (2004)	India	CC	Unclear	Yes	Sputum+ other	Some treated	FASTPlaque	103/129	BACTEC+LJ	0.94 (0.79,0.99	0.83 (0.52,0.98)
**In- house assays**											
McNerney (2004)	Zambia	CS	Yes	Yes	Sputum	NR	In-house	220/496	LJ	0.44 (0.37,0.51)	0.92 (0.89,0.95)
Mbulo (2004)	Zambia	CS	Yes	Yes	Sputum	Untreated	In – house	245/514	LJ+AMTD	0.45 (0.32,0.60)	0.95 (0.85,0.99)

Abbreviations: CS, cross-sectional studies; CC, case-control studies; NR, Not recorded.

Thirteen studies assessed the specificity of phage-based assays (Table 2, Figure 3). Specificity is the proportion of individuals without tuberculosis (culture-negative) who are negative by the phage- based assay. The specificity estimates were high and fairly consistent across the studies (range: 0.83 to 1.00). Except two studies [9,17] that reported false- positive rates of 14% and 17% respectively; all other studies reported >90% specificity. The in-house assays were as specific as commercials assays.

**Figure 4:**
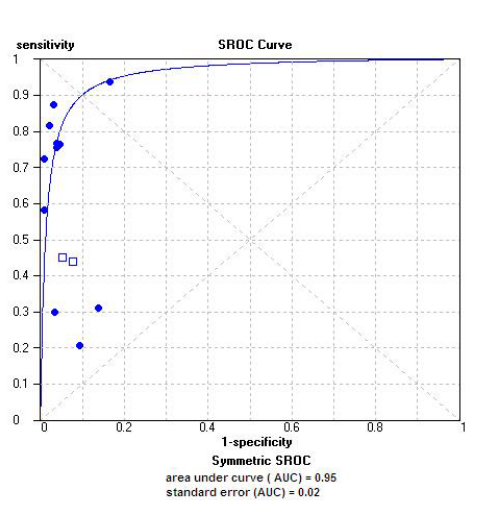
Summary receiver operating characteristic (SROC) curves for commercial and in-house assays. Each circle (commercial assay) and rectangle (in-house assay) represents an individual study.

Figure 4 presents the sensitivity and specificity estimates in a SROC space. The curve shows that most studies had high specificity with lower and highly variable estimates of sensitivity. Although the area under the curve was 0.95, significant heterogeneity in the sensitivity estimates precluded the determination of clinically meaningful summary estimates of accuracy.

### Effect of sputum smear status on accuracy of phage tests

**Figure 5:**
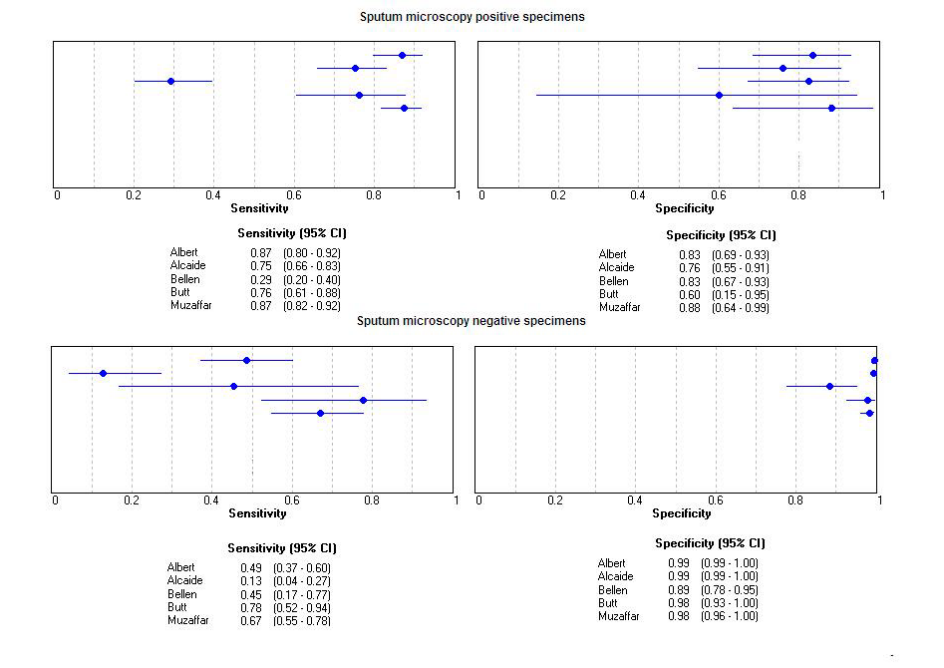
Forest plots of estimates of sensitivity and specificity for smear positive and smear negative specimens.

Because phage-based assays have threshold of detection of about 100 viable bacilli, they are expected to perform better in smear-positive samples compared to smear- negative ones. We performed subgroup analysis to evaluate this hypothesis. Five studies [5,6,8-10] provided estimates of accuracy, stratified by smear status (Table 3, Figure 5). Smear-positive specimens tended to yield higher estimates of sensitivity than smear-negative specimens. The sensitivity ranged between 0.29 and 0.87 in smear positive and 0.13 to 0.78 in smear negative specimens. The specificity ranged between 0.60 and 0.88 among smear positive specimens and 0.89 to 0.99 in smear negative specimens.

**Table 3:** Sensitivity and specificity of phage assays in studies that reported results stratified by smear microscopy status

			**Sputum smear microscopy status**			
			
			Smear positive		Smear negative	
			
Study	Country	Number of Specimens (culture positive)	Sensitivity	Specificity	Sensitivity	Specificity
Albert (2002)	South Africa	1618 (207)	0.87 (0.79,0.92)	0.83 (0.69,0.93)	0.49 (0.37,0.60)	0.99 (0.99,1.00)
Alcaide (2003)	Spain	2048 (144)	0.75 (0.66,0.83)	0.76 (0.55,0.91)	0.13 (0.04,0.27)	0.99 (0.99,1.00)
Bellen (2003)	Philippines	206 (103)	0.29 (0.20, 0.40)	0.83 (0.67,0.93)	0.45 (0.17,0.77)	0.89 (0.78,0.95)
Butt (2004)	Pakistan	160 (60)	0.76 (0.60, 0.88)	0.60 (0.15,0.95)	0.78 (0.52,0.93)	0.98 (0.93,1.00)
Muzaffar (2002)	Pakistan	514 (245)	0.87 (0.82,0.92)	0.88 (0.63,0.98)	0.67 (0.55,0.78)	0.98 (0,96,1.00)

### Head-to-head comparisons against sputum microscopy

**Figure 6:**
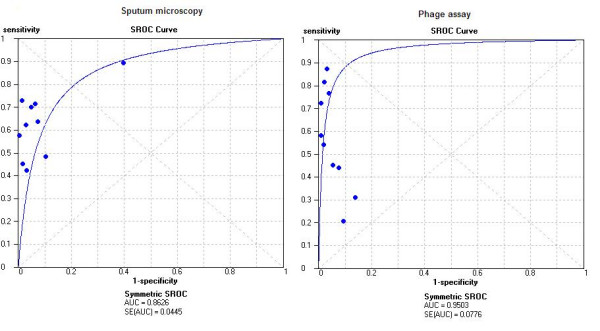
Summary receiver operating characteristic (SROC) curves for sputum microscopy and phage-based assays. Each solid circle represents a study in the meta-analysis.

Ten studies – 8 commercial [5-10,12,13] and 2 in-house [13,15] – provided data on head-to-head comparisons of sputum microscopy with phage based assays, against a common reference standard viz. culture [Table 4; Figure 6]. Eight studies reported staining sputum smears with Ziehl – Neelsen technique [6,7,9-12,16,17], whereas four studies used fluorescent microscopy [5,8,13,15]. Except three studies [5-7], other studies did not report clinically meaningful higher sensitivity for the phage assays. Interestingly, the sensitivity of phage based assays was lower than that of microscopy in four studies [8,9,13,15]. The SROC plots [Figure 6] suggest that phage assays have a slightly higher accuracy than microscopy (area under the SROC curve was 0.95 for phage assays, whereas it was 0.86 for microscopy).

**Table 4:** Head-to-head comparison between smear microscopy and phage assays

			Smear microscopy		Phage test		Difference (Phage – Microscopy)	
			
Study	Year	Sample size	Sensitivity	Specificity	Sensitivity	Specificity	Sensitivity	Specificity
**Commercial assays**								
Albay	2003	192	0.58 (0.44,0.70)	1.00 (0.97,1.00)	0.88 (0.77,0.94)	097 (0.92,0.99)	0.30	- 0.03
Albert	2002	1618	0.62 (0.55,0.69)	0.97 (0.96, 0.98)	0.72 (0.66,0.78)	0.99 (0.98,0.99)	0.10	0.02
Alcaide	2003	2048	0.73 (0.65,0.80)	0.99 (0.98,0.99)	0.58 (0.50,0.66)	0.99 (0.99,0.99)	-0.15	0
Bellen	2003	204	0.89 (0.82,0.95)	0.60 (0.50,0.70)	0.31 (0.22,0.41)	0.86 (0.78, 0.92)	- 0.58	0.26
Butt	2004	160	0.70 (0.57.0.81)	0.95 (0.89,0.998)	0.77 (0.64, 0.87)	0.96 (0.90,0.99)	0.07	0.01
Marei	2003	38	0.64 (0.31,0.89)	0.93 (0.76, 0.99)	0.55 (0.23, 0.83)	1.00 (0.87, 1.00)	-0.09	.07
Mbulo	2004	496	0.45 (0.32, 0.60)	0.98 (0.91, 1.00)	0.45 (0.32, 0.60)	0.91 (0.82, 0.96)	0	-.07
Muzaffar	2002	514	0.71 (0.65,0.77)	0,94 (0.90,0.96)	0.82 (0.76,0.86)	0.98 (0.95,0.99)	0.11	0.05

**In- house assays**								
Mbulo	2004	115	0.48 (0.29, 0.60)	0.90 (0.81, 0.95)	0.21,(0.08,0.40)	0.91 (0.82,0.96)	-0.27	0.14
McNerney	2004	496	0.60 (0.53,0.66)	0.87(0.83,0.91)	0.44 (0.37, 0.51)	0.92 (0.89,0.95)	- 0.16	0.05

## Discussion

### Overall accuracy of phage-based tests

Our results indicate that phage-based assays are highly specific but not sensitive enough to be equivalent to culture. Phage-based assays detected *M. tuberculosis *in one-half to two thirds of sputum samples with specificity that ranged between 0.83 and 1.00. On the other hand, sensitivities varied between 0.21 and 0.94. Ten of the 13 studies used FASTPlaque-TB kit; the two studies that used in-house assays were less accurate than the commercial assays.

Because of heterogeneity in estimates of accuracy across studies, we did not calculate pooled estimates of sensitivity and specificity. What factors might explain the observed heterogeneity? The differences in the extent of treatment given to patients prior to collecting specimens, the potential differences in timeliness of specimen transport and processing may have had impacts on the number of viable bacilli in the specimens. Since the phage-based assay detects only viable mycobacteria, and has a threshold of detection of about 100 viable bacilli, any factor that impacts viability of TB bacilli can affect sensitivity of the assay. Of the 5 studies of commercial assays in patients who were not treated prior to specimen collection, 4 studies reported sensitivities of 0.72 or greater. Conversely, only 2 of the 5 studies whose patient population included those on anti-TB therapy had sensitivities greater than 0.72. It is likely that anti-TB therapy in these studies decreased the number of viable bacilli in specimens. Further assessments of this issue should be made.

Another important issue that may have been responsible for heterogeneity in sensitivity estimates was specimen transport and the potential impact of environmental conditions on specimen viability. Rapid processing of specimens and timely initiation of the phage assay is important: the sensitivity of the phage assay was 72% when the specimens were processed immediately and tests were performed daily [5] compared to the sensitivity of 29% when the processing of the specimens was delayed and the assays were run twice a week [9]. The specificity of phage-assays for detecting acid fast bacilli is likely to decrease in settings in which infection with mycobacteria other than *M. tuberculosis *are common. Sodium hydroxide, used to decontaminate the specimens, may damage the acid fast bacilli and can reduce the sensitivity of phage-based assays for detecting tuberculosis. Use of gentler decontamination techniques can reduce the specificity of the test: this approach protects the acid fast bacilli but fails to prevent contamination from other microorganisms.

Contamination of LJ slants with micro-organisms is a common problem in certain settings (40.4% in Zambia [13] and 18.2% in Pakistan [6]). The exclusion of contaminated results may result in biased estimates of sensitivity and specificity. This approach (partial verification) can lead to bias if systematically more abnormal than normal test results are subjected to the reference standard. Although no study reported use of smear instead of contaminated cultures, such a strategy could weaken the reference standard and result in differential verification bias.

The impact of HIV infection on test accuracy could not be determined in this review because none of the studies reported the proportion of HIV infections among the tested population. Because HIV infection may impact the extent of viable mycobacteria present in sputum specimens, studies of populations with defined HIV status should be performed. Also, the varying sensitivity of the reference standard might have had an impact on the accuracy of the phage-based assays and could have contributed to the heterogeneity. Because grouping the studies in four sub-groups (LJ media, BACTEC media, LJ and BACTEC and AMTD and LJ) would have resulted in small numbers of studies in each of the category, we did not attempt statistical comparisons.

### Accuracy of phage-based tests compared to smear microscopy

Although some phage-based studies proved to be more sensitive than smear microscopy [5-7,10], others were not [8,9,12,13,15]. Differences in performance of phage-based tests compared to smear microscopy were also determined for smear-positive and smear-negative, culture-positive specimens. Overall, sensitivity in smear-positive specimens appeared to be higher than in smear-negative specimens, but among smear -negative specimens, phage- based assays had high specificity.

To evaluate the accuracy of smear microscopy for detecting *M. tuberculosis*, except one study [5] in which patients provided two sputum specimens each, all studies used only a single sputum sample. This approach differs from the current World Health Organization (WHO) and International Union against Tuberculosis and Lung Disease (IUATLD) recommendations, which state that at least three sputum samples must be examined for each patient [25]. Use of a single sample might make the smear microscopy look less sensitive than what it actually it is because the sensitivity may go up with greater number of smear tests. It would be interesting to evaluate the additional yield from repeated sputum examinations by microscopy and culture and do a head-do-head comparison of 3 sputum smears with phage-based assays. To our knowledge, this has not been done in any of the available studies.

Non- tuberculous mycobacteria are also known to contribute to false positive phage test results. The phage can be amplified by almost any mycobacteria present in the sputum and it is important to verify that the sputum contains *Mycobacterium tuberculosis *complex. The effect of non-tuberculosis mycobacteria (NTM) might be minimal in places where true *M. tuberculosis *is very common (high incidence settings). In settings with low incidence, NTM might have a greater impact. A requirement for a confirmatory test would make the overall testing strategy more expensive, delay the diagnosis, and result in an additional patient visit to the laboratory. Clearly, these factors could adversely impact the practical applicability of the test in resource -limited settings with high burden of tuberculosis.

Spectrum and selection bias are known to affect sensitivity and specificity of a diagnostic test. Spectrum bias may occur when the test is assessed in a study population with a different clinical spectrum than will be found among those in whom test is to be applied in clinical practice. Specimens in several reviewed studies were collected from patients reporting to tertiary care centres [10,11,16,17]. Such patients are more likely to have advanced disease with a large bacillary load. Selection bias could also have played a role in influencing the diagnostic properties of phage-based assays – patients referred to chest clinics and those with abnormal chest radiographs are more likely to have a higher bacillary load. The referral bias can make the phage-based assays appear more sensitive than they actually are.

Overall, our review suggests that when a patient's phage test is negative, there is roughly one in three probability that patient has tuberculosis. A negative test, therefore, does not exclude tuberculosis in patients suspected to have tuberculosis.

### Strengths and weaknesses of the review

Our review has several strengths. We followed a written protocol, performed a very comprehensive search of several databases and sources to identify studies. We assessed the quality of included studies by using established criteria [20]. We also analyzed commercial and in-house assays separately, to take into account the potential differences between the assay techniques. Lastly, we used summary ROC curves to take into account the impact of varying thresholds and also the interdependence of sensitivity and specificity.

Our review has some limitations. Most studies provided no data on prevalence of TB and/or HIV in their study population. Several studies did not specify whether the specimens were collected before or after anti-TB therapy was started. Most studies did not specify the volume and quality of sputum specimens, the proportion of contaminated phage tests and indeterminate results. Most studies did not specify if non-tuberculous mycobacteria were isolated in the study. We could not analyse such factors as laboratory infrastructure and expertise with phage assays on the accuracy of phage assays. Although we explored the issue of heterogeneity using subgroup analyses, techniques such as meta-regression may be useful in the evaluation of heterogeneity. However, we were unable to perform metaregression because we had only 13 studies in our systematic review; with only 13 data points, it is difficult to fit and interpret a regression model. Also, because of the small number of data points, we were unable to perform statistical comparisons between subgroups. Finally, bias may have been introduced by the exclusion of non-English language studies.

### Clinical applicability and implications

Since many countries with a high TB burden do not use culture, an inexpensive alternative diagnostic test is necessary. One-third to two-thirds of all cases of pulmonary tuberculosis are not being detected by the commonly used smear microscopy [25]. Can phage-based tests be used to diagnose pulmonary tuberculosis in high burden and limited resource settings? Although our review shows that phage-based tests have high specificity, their sensitivity is lower and highly variable. Also, they do not have substantially higher accuracy than sputum microscopy. In addition, phage-based assays are much more complicated and labor-intensive than microscopy and thus cannot be performed in primary-care settings; they require a laboratory infrastructure similar to that required for performing cultures.

Although it can be argued that the rapid and specific diagnosis of 50% or more of TB patients can be made within 2 days, leading to reduced potential for ongoing transmission and more effective management of individual patients [26], our review suggests that phage-based assays need to have higher sensitivity before such potential advantages can be realized. Mbulo et al [13] point out that the main disadvantage of phage-based test compared to sputum smear microscopy is that specimens need to be transported to a specialist reference laboratory. The fact that phage-based assays are more expensive than smear microscopy, need a trained microbiologist makes them less suitable in resource-poor settings. Also, it remains to be seen how the test performs outside reference laboratories. Molecular strain typing tests such as polymerase chain reaction (PCR) are competitors for phage-based assays. Of the two studies [7,12] that reported head to head comparisons of molecular tests with phage – based assays against a common reference test (i.e. culture), one study [7] found no difference in test accuracy between the phage-based assay and the polymerase chain reaction; the other study [12] found that phage-based assay was less sensitive (64% vs. 82%) but more specific (93% vs. 85%) compared to the polymerase chain reaction.

## Conclusion

Our review suggests that phage-based assays have high specificity, but modest and variable sensitivity. Their accuracy is slightly higher than smear microscopy in head to head comparisons. However, because of the overall low sensitivity, the similarity of phage-based assays to sputum microscopy with respect to accuracy, and the need for a fairly advanced laboratory infrastructure, phage-based assays cannot replace conventional diagnostic tests at this point. Further research is required to identify methods that can enhance the sensitivity of phage-based assays, without compromising the high specificity.

## Competing interests

The author(s) declare that they have no competing interests.

## Authors' contributions

SK formulated the research question, searched the databases, extracted the data, performed the statistical analysis and wrote the manuscript. MP participated in the study design, extracted a part of data, and helped to draft the manuscript. LP contributed to the development of the study protocol, provided critical inputs in laboratory associated issues and reviewed the manuscript. LR and AR contributed to the review of the manuscript. All authors read and approved the final manuscript.

## Ethical approval

Not required.

## Pre-publication history

The pre-publication history for this paper can be accessed here:



## Supplementary Material

Additional File 1 Permission to reproduce figure 1 from *Biomedica*. Click here for file
