# Supplementary material for: Bacteriophage- based tests for the detection of Mycobacterium tuberculosis in clinical specimens: a systematic review and meta- analysis
Source: BMC Infect Dis. 2005 Jul 16;5:59. doi: 10.1186/1471-2334-5-59 (PMC1180828; doi:10.1186/1471-2334-5-59)
Supplement: Additional File 1 — Permission to reproduce figure 1 from Biomedica. [file 1471-2334-5-59-S1.pdf]

## Madhukar Pai

**From:** Carlos Arturo Hernandez [cahernandez@ins.gov.co]
**Sent:** Tuesday, December 28, 2004 7:34 AM
**To:** madhupai@berkeley.edu
**Cc:** ecastaneda@ins.gov.co
**Subject:** Re: Permission to reproduce figures from Biomedica
**Importance:** High

Dear Dr. Pai,
On behalf of the Editorial Committee of *Biomédica* I would like to let you know that the Committee has granted permission to reproduce figures 5 and 6 of the article **Recent advances in molecular methods for early diagnosis of tuberculosis and drug-resistant tuberculosis.** Biomédica 2004;24(Supp.1):149-62.
Please, let us know as soon as your submitted articles had been accepted for publication.
Sincerely yours,
Carlos Arturo Hernández
Executive editor, *Biomédica*

----- Original Message -----
**From:** Elizabeth Castañeda
**To:** Biomédica; Carlos Arturo Hernandez
**Sent:** Thursday, December 23, 2004 5:19 PM
**Subject:** RV: Permission to reproduce figures from Biomedica

Elizabeth Castañeda, Ph.D.
Subdirección de Investigación
Instituto Nacional de Salud
Avenida calle 26 N° 51-60
Tel: 220-09-20
Fax: 220-7700 Ext. 255

---

**De:** Madhukar Pai [mailto:madhupai@berkeley.edu]
**Enviado el:** Jueves, 23 de Diciembre de 2004 12:50 p.m.
**Para:** ecastaneda@ins.gov.co
**Asunto:** Permission to reproduce figures from Biomedica

Dr Elisabeth Castaneda
Editor, Biomedica

Dear Dr Castaneda:

I am a postdoctoral fellow at the University of California, Berkeley & San Francisco. I am writing to you at the suggestion of Dr Manzour Hazbon, whose recent review article was published in your journal: **Recent advances in molecular methods for early diagnosis of tuberculosis and drug-resistant tuberculosis.** Biomedica. 2004 Jun;24 Supp 1:149-62.

This excellent review article has two figures (Figures 5 and 6) that we would like reproduce, with your permission, in two meta-analyses our group at Berkeley has conducted: on the accuracy of phage-based assays for TB diagnosis, and for the detection of drug resistance. These manuscripts are likely to be submitted for publication to *BioMed Central Infectious Diseases*, and another journal (yet to be decided). I will be sure to inform you of the journal details as soon as we finalize it.

Please do give us the permission to reproduce the figures. We will, needless to say, provide the full citation of the paper in our manuscript and state that they have been reproduced with permission from Biomedica. Dr Hazbon, the author, has already given us his permission to use the figures.

Thanks in advance,

With best regards,

Madhu

Madhukar Pai, MD, PhD
Postdoctoral Fellow
Div. of Epidemiology, UC Berkeley
Div. of Pulm & Crit Care Med, SFGH, UCSF

Mailing address:
School of Public Health
140 Warren Hall
Berkeley, CA 94720
Tel: 510-388-7137
Fax: 510-643-4927
Email: madhupai@berkeley.edu
**URL: www.medepi.net/meta**
